# Bringing back *Galium aparine* L. from forgotten corners of traditional wound treatment procedures: an antimicrobial, antioxidant, and in-vitro wound healing assay along with HPTLC fingerprinting study

**DOI:** 10.1186/s12906-024-04355-y

**Published:** 2024-07-23

**Authors:** Amirreza Dowlati Beirami, Negin Akhtari, Razieh Noroozi, Dara Hatamabadi, Syed Muhammad Farid Hasan, Seyed Abdulmajid Ayatollahi, Neda Alsadat Ayatollahi, Farzad Kobarfard

**Affiliations:** 1 Department of Medicinal Chemistry, School of Pharmacy, Shahid Beheshti University of Medical Sciences, PO Box: 14155-6153, Tehran, Iran; 2 Department of Pharmaceutics and Pharmaceutical Nanotechnology, School of Pharmacy, Shahid Beheshti University of Medical Sciences, Tehran, Iran; 3 Division of Food Safety and Hygiene, Department of Environmental Health Engineering, School of Public Health, Tehran University of Medical Sciences, Tehran, Iran; 4 Iranian Food and Drug Administration, Ahvaz Jundishapur University of Medical Sciences, Ahvaz, Iran; 5 Department of Pharmaceutics, Faculty of Pharmacy & Pharmaceutical Sciences, University of Karachi, Karachi, Pakistan; 6 Phytochemistry Research Center, Shahid Beheshti University of Medical Sciences, Tehran, Iran

**Keywords:** Traditional medicine, Phytochemicals, GA, *Galium aparine L.*, Wound healing, Antibacterial assay, Antioxidant activity

## Abstract

**Background:**

The wound healing process, restoring the functionality of the damaged tissue, can be accelerated by various compounds. The recent experimental analysis highlights the beneficial effects of phytochemicals in improving skin regeneration and wound healing. In traditional medicine, one of the widespread plants used for treating different injuries or skin afflictions is *Galium aparine L*. (GA). Besides, previously reported chemical compounds of GA suggested its therapeutic effects for the wound healing process, yet its regulatory effects on the cellular and molecular stages of the wound healing process have not been investigated.

**Methods:**

In the present study, the phytochemical profile of the GA extract was analyzed using HPTLC fingerprinting, and further scientific evaluation of its phytochemicals was done. The wound-healing effects of GA extract were explored at the cellular and molecular levels while accounting for cell toxicity. The wound closure enhancing effect, antibacterial activity, and antioxidant activity were assessed.

**Results:**

The HPTLC fingerprinting of the GA extract proved its previously reported phytochemical profile including phenols, flavonoids, tannins, plant acids, ergot alkaloids, flavonoids, anthraquinones, terpenoids, sterols, salicin, lipophilic compounds, saponins, iridoids, and heterocyclic nitrogen compounds. Antimicrobial assessment, of the extract, indicated the more susceptibility of *S. aureus* to the inhibitory effects of GA rather than *E. coli* and *S. epidermidis*. DPPH test results revealed the antioxidant property of GA extract, which was comparable to ascorbic acid. The results of the viability assay showed no cytotoxicity effects on human umbilical endothelial cell (HUVEC) and normal human dermal fibroblast (NHDF) cell lines treated with different concentrations of whole plant extract and cell viability increased in a dose-dependent manner. The results of the scratch assay showed improved cell migration and wound closure.

**Conclusions:**

This study shows the anti-oxidant, anti-microbial, and in vitro wound healing wound-healing effects of GA hydroalcoholic extract, which aligns with its use in traditional medicine. No cytotoxicity effects were shown. The results from this study can be the basis for further investigations such as animal models and phytochemical studies. Further evaluation of its effects on mechanisms and signaling pathways involved in the wound healing processes such as angiogenesis and cell proliferation can provide novel insights into the potential therapeutic effects of the GA extract.

## Introduction

A wound is a disruption of healthy anatomic structure and function of living tissues. Therefore, wound healing can be defined as the process that results in restoring anatomic continuity and function of disrupted tissue [1, 2]. The process of wound healing can be divided into three overlapping stages: the inflammatory stage, the proliferative stage, and the remodeling stage. The inflammatory stage begins with hemostasis and is followed by the chemotaxis of inflammatory cells. The damaged blood vessels contract in hemostasis, and the leaked blood starts to coagulate to prevent further blood loss. The activation of the coagulation cascade and clot formation results in the release of mediators and chemokines from thrombocytes [3]. CXCL8 is a potent neutrophil attractant chemokine released by platelets and chemokines such as CCL2, CCL3, and CCL5, released by neutrophils recruit macrophages [4]. Macrophages play an important role in phagocyting debris, apoptotic neutrophils, and bacteria in the wound site to sterilize and prepare for tissue regrowth [5]. 2–4 days after wounding, they become major inflammatory cells in the wound for approximately 14 days. Macrophages protect the wound from invading microorganisms and release growth factors, cytokines, and chemokines such as TGF-β, VEGF, and bFGF to promote cellular proliferation and migration [4]. The remodeling phase, the latest and prolonged phase, occurs after the closure of the wound to reorganize the extracellular matrix (ECM). During the proliferative phase, ECM molecules deposit in a disorderly manner, degrade, resynthesize, and cross-link in the remodeling phase to achieve the maximum possible tensile strength of the incision wound [3, 6]. In this phase, enzymes such as matrix metalloproteinases (MMPs) are responsible for ECM degradation [7]. On the other hand, metalloproteinase tissue inhibitors (TIMPs) act as inhibitors of MMPs and have an essential role in regulating the ECM remodeling process [8].

Active ingredients can promote these processes. These ingredients used in wound healing can be synthetic or derived from natural sources (extracts from plants, microorganisms, and animals). Multiple in-vitro and in-vivo models can assess the potential of active ingredients in wound healing. The most common in-vitro wound healing models are cell viability and scratch-wounding cell migration assay performed on cell models. On the other hand, in-vivo models of wound healing, such as superficial wounds and burn wounds, are performed on mammalian models [9, 10].

The beneficial effect of plants in wound healing has been shown and reported in many scientific studies. Compared to chemical agents, generally, plants have lower costs and are accessible and reliable resources of medicinal substances. Also, they exhibit fewer side effects making them more cost-effective therapies in wound healing [11–14].

*Galium aparine L. (GA),* also known as cleavers and Catchweed Bedstraw, is a fast-growing herbaceous plant of the family Rubiaceae. It is a common plant growing in hedgerows and on the waste ground [15, 16]. Traditionally, GA is used as a diuretic to relieve bloating, but it has also been used for other problems such as lymph swellings, jaundice, fever, hypertension, and wounds. Also, as a folk medicinal recipe, the ointment-like consistency made from butter and GA fresh juice is used for scalds, burns, and swelling [17]. Furthermore, the results of a study on the antioxidant activities of GA indicate that an aqueous fraction of GA’s methanolic extract exhibits considerable antioxidant activity [18].

The wound-healing process can be retarded due to different factors such as overproduction of reactive oxygen species (ROS), bacterial infection, vascular insufficiency, and diabetes [19, 20]. In this research, we assessed the potential effect of GA extract in the wound healing process.

## Material and methods

### Plant material

Although laws of “environmental protection and improvement” in Iran and national guidelines do not cover the collection of plant materials for small research, institutional permissions are often required for collecting plant material. As a result, permissions for collecting GA were obtained from the Shahid Beheshti University of Medical Sciences on 18 February 2018 The GA whole plant was collected from a mountainous region of Tehran province, Darakeh, Iran, in April 2019. Consequently, the authentication of the plant species was established by the botanist of Shahid Beheshti University of Medical Sciences, Mohammad Kamalinejad.

Voucher specimen for *Galium Aparine L.* has been deposited in the herbarium of Shahid Beheshti University of Medical Sciences as well as many other public herbariums such as the herbarium of Damghan University [21], and the herbarium of Kermanshah university [22].

### Extract preparation

The maceration method was applied for plant extraction, slightly modifying the previous experimental method [18]. The cleaned plant was washed with tap water, dried well, and ground with an industrial grinder to obtain a homogenized powder. 75 g of the prepared powder was macerated into 50% hydroethanolic solvent and shaken well on a shaker (150 rounds per second) at room temperature. After 72 h, the outcome was filtered using a simple filter paper and vacuum filtration; Remaceration of the residue was conducted in duplicate. Subsequently, the extract was evaporated in a vacuum rotary evaporator at 40 °C; finally, the residue was stored at 4 °C for further investigation.

### Phytochemical fingerprinting by high-performance thin-layer chromatography (HPTLC)

To establish the unique fingerprint phytochemical pattern of the extract, a total of 0.02 g of hydroalcoholic extract was loaded on HPTLC Plates Silica gel 60 F 254 (Merck, Germany) using an Automatic TLC sampler (ATS4, CAMAG). According to the phytochemical groups targeted to be identified, specific mobile phases and derivatization reagents were utilized. Saturation time, humidity, migration distance, and drying time were adjusted based on the standard operating procedure for HPTLC [23–25]. Chromatograms were observed under visible, UV (254 nm wavelength), and UV (366 nm wavelength) before and after derivatization. Documentation was carried out by TLC visualizer and winCATS software, version 1.4.4.

- To recognize phenols, flavonoids, tannins, plant acids, and ergot alkaloids, the spotted plates were developed in the respective mobile phase toluene – acetone – formic acid (4.5:4.5:1). Derivatization was performed using ferric chloride solution reagent [26, 27].
- For anthraquinones identification and flavonoids confirmation, the mobile phase was toluene – acetone – formic acid (4.5:4.5:1) as well, but ethanolic aluminum chloride reagent was utilized as a derivatization reagent [26, 28].
- For terpenoids, sterols, salicin, ergot alkaloids, and lipophilic compounds identification, n-hexane – ethyl acetate (7:3) was used as mobile phase, and derivatization reagent was vanillin phosphoric acid [26, 29, 30].
- For terpenoids, sterols, and most lipophilic compounds, confirmation, n-hexane – ethyl acetate (7:3) as mobile phase and anisaldehyde–sulphuric acid derivatization reagent were used. Also, saponin and iridoid recognition was confirmed by these reagents as well [26].
- Alkaloids and heterocyclic nitrogen compounds identification were conducted using chloroform-methanol (8:2) as the mobile phase and derivatization was performed using Dragendorff reagent [26].
- To confirm the presence of alkaloids and heterocyclic nitrogen compounds, the mobile phase was chloroform-methanol (8:2), and ethanolic sulphuric acid reagent was applied for derivatization, and chromatograms were observed before and after derivatization [26].

The summary of mobile phases and derivatization reagents is shown in Table 1. Each of the six plates was observed under visible, and UV (254 nm and 366 nm) before and after derivatization.

**Table 1:** Mobile phase and derivatization reagents list for HPTLC fingerprint profiling of *Galium aparine* L. extract

Plate No.	Mobile phase	Derivatization reagent
1	Toluene – acetone – formic acid (4.5:4.5:1)	Ferric chloride solution regent
2	Toluene – acetone – formic acid (4.5:4.5:1)	Ethanolic aluminum chloride reagent
3	n-hexane – ethyl acetate (7:3)	Vanillin phosphoric acid reagent
4	n-hexane – ethyl acetate (7:3)	Anisaldehyde sulphuric acid reagent
5	Chloroform – methanol (8:2)	Dragendorff reagent
6	Chloroform – methanol (8:2)	Ethanolic sulphuric acid reagent

### Minimum inhibitory concentration (MIC) test

To assess the antibacterial activity of GA extract on *Staphylococcus aureus, Escherichia coli, and Staphylococcus epidermidis* species, the MIC value was determined by the broth dilution method [31]. A concentration of 400 μg/ml was prepared by dissolving the hydroalcoholic extract in DMSO 5%, subsequently, serial dilutions of the extracts were prepared in micro-titer wells filled with 100 μL of Mueller–Hinton broth to cover the concentration range of 12.5–400 μg/ml. 100 μL of bacterial suspension (10^6^ cells/mL) was added to each well and eventually incubated at 37 °C for 24 h. DMSO 5% was applied as the control.

### Inhibition zone assessment

The disc diffusion method for antimicrobial susceptibility assessment was performed according to the previous experiments [32, 33]. In this process, *Staphylococcus aureus* ATCC 25923 and *Escherichia coli* ATCC 25922 adjusted with 0.5 McFarland Turbidity standard were cultured separately in Mueller Hinton Agar [34]. A solution of GA in 2% DMSO with a concentration of 800 mg/ml was prepared and diluted with water to achieve concentrations of 400 mg/ml and 200 mg/ml. Subsequently, four wells with a diameter of 8 mm were punched into the agar mediums and filled with 100 μl of the extract with concentrations of 800 mg/ml, 400 mg/ml, and 200 mg/ml. Also, the fourth well was filled with the same volume of 2% DMSO as the negative control. Plates were then incubated at 37 °C for 24 h, and finally, inhibition zone diameters were measured with caliper

### Antioxidant assay

DPPH test was conducted to evaluate the antioxidant activity of GA [26, 27]; pursuing this goal, serial dilutions were prepared with a 200–800 mg/ml concentration range. A stock solution of DPPH was provided by dissolving 2, 2-diphenyl-1-picrylhydrazyl (DPPH) in methanol to reach a 0.1 mM concentration. Eventually, 100 μL of DPPH solution was added to each well. Ascorbic acid was employed as an antioxidant reference in the same volume and concentration. After 30 minutes of wells stored in dark conditions at room temperature, The DPPH absorbance was valued at the wavelength of 517 nm. The formula below was applied to indicate the radical scavenging function of samples:

$$\textrm{DPPH}\ \textrm{scavenging}\ \textrm{activity}\ \left(\%\right)=\left[\left(\textrm{A}0-\textrm{A}1\right)/\textrm{A}0\right]\times 100.$$

Where A0 is the absorbance value at 517 nm of the methanolic solution of DPPH (negative control), and A1 is the absorbance value at 517 nm for the sample. In this experiment, samples of GA extract and Ascorbic acid were prepared in triple repeats.

### Culture of human umbilical endothelial cell and Normal human dermal fibroblasts

HUVEC and NHDF were obtained from the Pasteur Institute of Tehran, Iran. The cell culture procedure was conducted based on the cell culture protocol [35]. Cells were observed under a phase-contrast microscope to ensure the cells were healthy and free of contamination. Both cells were cultured in a 96-well plate containing Dulbecco’s Modified Eagle Medium (DMEM; Sigma), and the process was followed by adding a 15% (v/v) inactivated FBS (Sigma). Also, 1% (v/v) antibiotic/antimycotic solution (AB/AM; Sigma) was utilized to protect the medium from bacterial and fungal interaction. Finally, both cultured cell lines were incubated in standard conditions (37 °C and 0.5% CO2) for 24 h.

### Cell viability and MTT assay

To evaluate the effect of whole plant extracts on cell viability, an MTT assay was conducted in multi-well microplates as explained elsewhere [36]. For this purpose, cultured cells were treated with a concentration range of 12.5 mg/ml – 400 mg/ml of the extract for 24, 48, and 72 h. Then, cells were incubated with 3-(4,5-dimethylthiazol-2-yl)-2,5- diphenyl tetrazolium bromide for 24 h. The viable cells will be penetrated readily by the MTT solution which is positively charged, and changing the soluble MTT into colored insoluble formozan as the result. Dimethyl sulfoxide (DMSO) with 0.03125, 0.0625%. 0.125, 0.25, 0.5, 1% concentration was applied as a control sample. Finally, after the color change, the absorption was valued by a 96-well plate reader. Based on the MTT assay protocols and equation below, cell viability was assessed.

$$\textrm{Viability}\ \left(\%\;\textrm{of}\;\textrm{sample}\right)=\left[\left(\textrm{Abs}\ \textrm{sample}\_\textrm{Abs}\ \textrm{blank}\right)/\left(\textrm{Abs}\ \textrm{control}\_\textrm{Abs}\ \textrm{blank}\right)\right]\ast 100.$$

In this formula, Abs sample, Abs control, and Abs blank represent samples’ absorbance, the absorbance of culture media +DMSO, and the absorbance of culture media without treatment, at 570 nm, respectively.

### In vitro wound-healing assay

The proliferation capability of NHDF and HUVEC treated with GA extract was evaluated using a scratch wound assay based on the approved protocol, which estimates the expansion of the cell population in an area [37]. In this regard, cultured NHDF and HUVEC cells were transferred to a 12-well plate (120,000 cells/ well). To achieve 70–80% confluence, cells were incubated with DMEM and 10% FBS for 24 h. Afterward, a starvation condition with 1% FBS was provided to eliminate the growth factor effect on cell proliferation. Then, culture media containing 10% FBS was supplemented. A linear scratch was generated with a sterile micropipette. 100 μL of the extract (50 and 400 mg/mL), allantoin (as positive wound healing control), and DMSO (1 and 0.125% v/v) were tested. The representative images in Figs. 7 and 8 were photographed at 0, 6, 12, 24, and, 48 h by the image-j software to peruse the relative migration cells. Wound closure calculation was performed employing this formula:

$$\textrm{Wound}\ \textrm{closure}\ \left(\%\right)=\left[\left({\textrm{A}}_0-{\textrm{A}}_{\textrm{h}}\right)/{\textrm{A}}_0\right]\times 100.$$

In the formula above, A_0_ is the primary, and A_h_ is the secondary wound (scratch) area.

### Statistical analysis

The areas of scratches were manually measured by Image J/Fiji® software. To compare the difference between the areas of scratch in wound healing scratch assay, we used two-way ANOVA and, Tukey tests to perform multiple comparisons, and *P* values less than 0.05 were considered statistically significant. Analyses were performed by Graph Pad Prism software version 9.0.0. Other statistical demonstrations in this study were prepared using MS Excel 2021.

## Result

### HPTLC

**Fig. 1:**
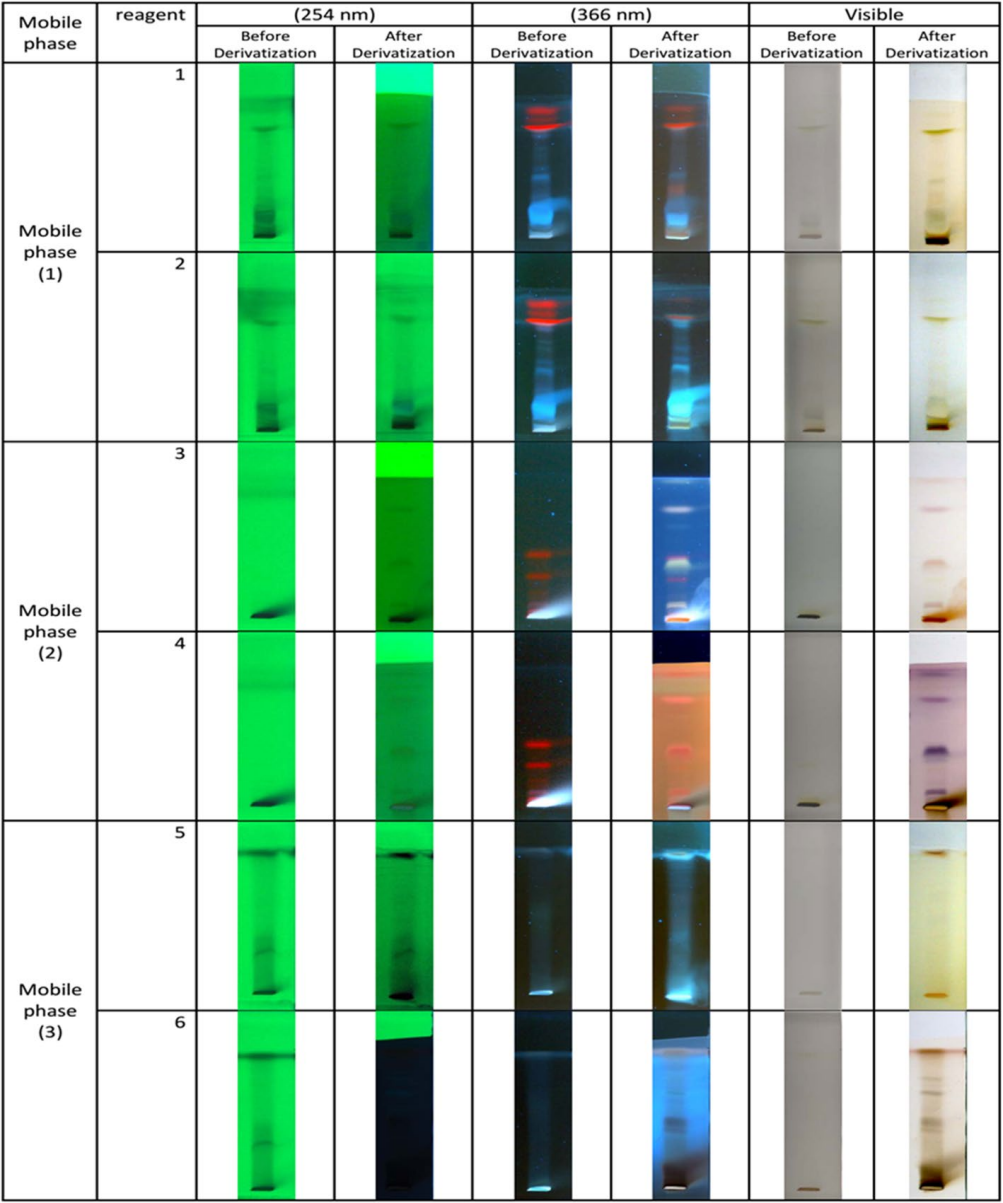
HPTLC fingerprint profile of hydroethanolic extract of *Galium aparine* L

The results from HPTLC profiling are shown in Fig. 1. In this figure, yellow and gray-colored spots that appeared after derivatization with ferric chloride reagent under visible light generally indicate the presence of tannins, phenols, flavonoids, and ergot alkaloids. Flavonoids and anthraquinones are presented after derivatization by ethanolic aluminum chloride reagent under UV light with a wavelength of 366 nm. The presence of blue spots in the plate derivatized by Vanillin phosphoric acid indicates the presence of terpenoids and most lipophilic compounds in the extract. For the detection of alkaloids, Dragendorff reagent and ethanolic sulphuric acid reagent were used to represent alkaloids in orange-brown and yellowish colors, respectively. Finally, the violet spots that appeared after derivatization by anisaldehyde sulphuric acid reagent can indicate the presence of glycosides, saponins, and terpenoids.

### MIC assay

According to the broth micro dilution method result, the lowest MIC of *GA* extract was related to *S. aureus* with a value of 50 mg/ml. MIC value against both *E. coli* and *S. epidermidis* was 100 mg/ml. Accordingly, *S. aureus* was inhibited in a lower extract concentration and revealed more susceptibility than two other species. However, *E. coli* and *S. epidermidis* responded almost the same.

### Assessment of inhibition zone diameter

GA extract exhibited antimicrobial activity, as displayed by the growth inhibition zones in Table 2. The largest zone of growth inhibition referred to *S. aureus*, which indicates it to be more susceptible than *E. coli*. Higher inhibitory activity was perceived as the extract concentration increased, so the dose-dependent antibacterial activity of GA extract may be concluded. The results obtained from this test tie well with MIC value determination, indicating *S. aureus* as the most susceptible bacteria to GA extract. No inhibition zone was observed for DMSO as the negative control.

**Table 2:** Inhibition zone assessments (IZ) results for *Galium aparine* L. extract against S.aureus, E.coli, and S.epidermidis

Extract Concentration	E.coli IZ	S.aureus IZ
50 mg/ml	1.34 cm	1.79 cm
100 mg/ml	1.70 cm	2.02 cm

### Antioxidant assay

**Fig. 2:**
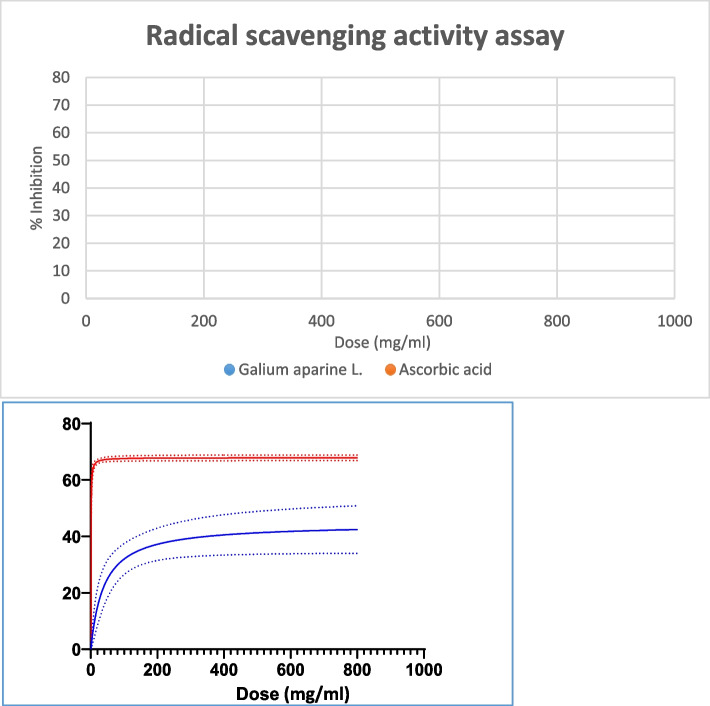
Radical scavenging activity of *Galium aparine* L. extract and ascorbic acid solution by DPPH method

The antioxidant activity of GA extract was assessed in this research according to DPPH reduction. The results demonstrated the antioxidant property and radical scavenging capability of GA extract. The highest antioxidant activity was observed in 200 mg/ml, and in lower concentrations, GA radical scavenging occurred in a dose-dependent manner. GA extract antioxidant activity was confirmed in this experiment, but ascorbic acid exhibited higher radical scavenging activity in all concentrations (Fig. 2).

### MTT assay

**Fig. 3:**
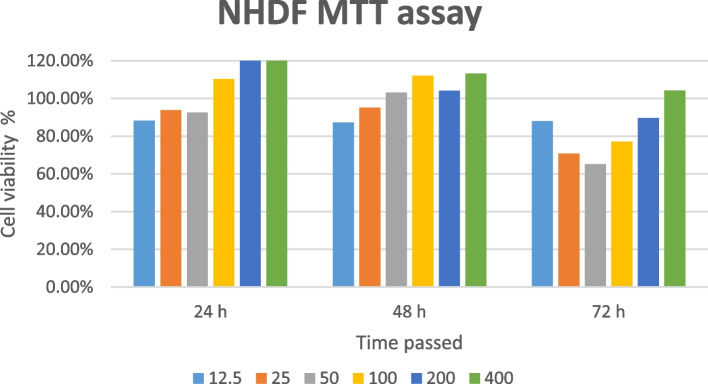
Cell viability indicated by the results of MTT assay of NHDF cell line exposed to *Galium aparine* L. extract

**Fig. 4:**
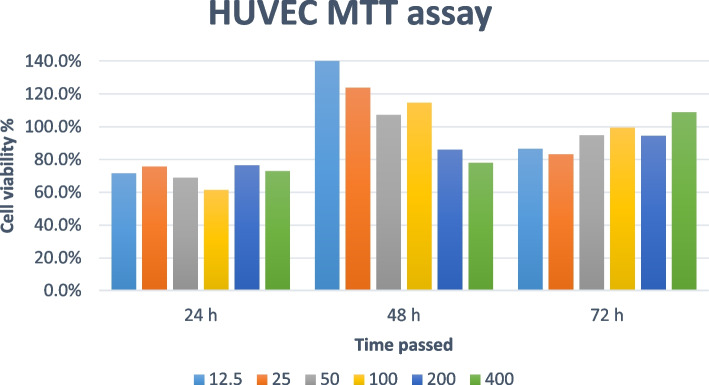
Cell viability indicated by the results of MTT assay of HUVEC cell line exposed to *Galium aparine* L. extract

MTT assay was performed to assess HUVEC and NHDF cell line viability exposed to GA whole plant extract. Figures 3 and 4 exhibit the admissible viability of NHDF and HUVEC cell lines treated with GA extract, respectively. Interestingly, in some concentrations, GA extract did not cause toxicity to the treated cells and increased cell viability.

In NHDF cells exposed to GA extract, in some periods a slight increase was observed in cell viability as the extract concentration was increased. The highest NHDF cell viability was observed during the first 24 hours in the cells treated with 400 mg/ml of GA extract. Higher viability was observed in the HUVEC cell line exposed to GA as the concentration increased during the first and third days. However, on the second day, cell viability decreased in higher concentrations. The highest HUVEC viability was perceived after 48 h with the 12.5 mg/ml concentration. Besides perceived evidence, additional experiments may be required to determine the range of concentration as the safe dosage.

### In vitro wound healing assay

**Fig. 5:**
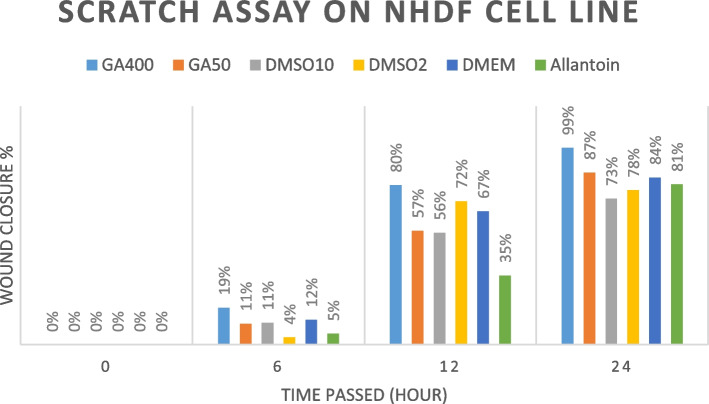
Results of the scratch assay on NHDF cells line treated by *Galium aparine* L. (400 mg/ml and 50 mg/ml), DMSO 2% (as negative control), DMEM (as positive control), and Allantoin (50 mg/ml)

**Fig. 6:**
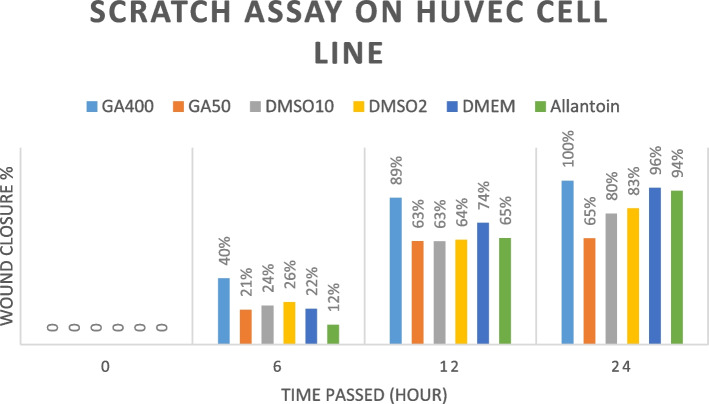
Results of the scratch assay on the HUVEC cells line treated by *Galium aparine* L. (400 mg/ml and 50 mg/ml), DMSO 2% (as negative control), DMEM (as positive control), and Allantoin (50 mg/ml)

Figures 5 and 6 demonstrate the impact of GA extract on cell migration and in vitro wound closure. In both cell lines, 400 mg/ml GA extract exhibited the highest potency in wound healing, and its healing capability increased over time. Both 400 mg/ml and 50 mg/ml concentrations of GA were more effective than allantoin as a well-known wound healing agent in NHDF and HUVEC cell lines. In this case, 400 mg/ml of GA extract reaches nearly 100% effects in 24 hours in both cell lines. It is noteworthy that unlike 400 mg/ml of GA, wound healing effects of allantoin was insignificant until 12 hours after the treatment in both cell lines.

The statistical analyses were shown in Tables 3 and 4 for the NHDF cell line and HUVEC cell line, respectively.

**Table 3:** The statistical results of wound healing assay on NHDF cell line

Source of Variation	% of total variation	*P* value		*P* value summary	Significant?
Row Factor	94.12	< 0.0001		****	Yes
Column Factor	2.719	0.0709		ns	No
Tukey’s multiple comparisons test	**Mean Diff.**	**95.00% CI of diff.**	**Below threshold**	**Summary**	**Adjusted** ***P*** **Value**
GA50 vs. GA400	10.75	−7.608 to 29.11	No	ns	0.4372
GA50 vs. DMSO10	14.50	−3.858 to 32.86	No	ns	0.1662
GA50 vs. DMSO2	11.00	−7.358 to 29.36	No	ns	0.4137
GA50 vs. DMEM	8.750	−9.608 to 27.11	No	ns	0.6410
GA50 vs. Allantoin	19.25	0.8922 to 37.61	Yes	*	0.0373
GA400 vs. DMSO10	3.750	−14.61 to 22.11	No	ns	0.9834
GA400 vs. DMSO2	0.2500	−18.11 to 18.61	No	ns	> 0.9999
GA400 vs. DMEM	−2.000	−20.36 to 16.36	No	ns	0.9991
GA400 vs. Allantoin	8.500	−9.858 to 26.86	No	ns	0.6669
DMSO10 vs. DMSO2	−3.500	−21.86 to 14.86	No	ns	0.9878
DMSO10 vs. DMEM	−5.750	−24.11 to 12.61	No	ns	0.9049
DMSO10 vs. Allantoin	4.750	−13.61 to 23.11	No	ns	0.9548
DMSO2 vs. DMEM	−2.250	−20.61 to 16.11	No	ns	0.9984
DMSO2 vs. Allantoin	8.250	−10.11 to 26.61	No	ns	0.6926
DMEM vs. Allantoin	10.50	−7.858 to 28.86	No	ns	0.4614

The symbols * and **** are used to demonstrate the level of significance, indicating *P* values less than 0.05 and 0.0001, respectively

**Table 4:** The statistical results of wound healing assay on HUVEC cell line

Source of Variation	% of total variation	*P* value		*P* value summary	Significant
Row Factor	94.10	< 0.0001		****	Yes
Column Factor	3.124	0.0307		*	Yes
Tukey’s multiple comparisons test	**Mean Diff.**	**95.00% CI of diff.**	**Below threshold?**	**Summary**	**Adjusted** ***P*** **Value**
GA50 vs. GA400	−20.00	−37.26 to − 2.744	Yes	*	0.0189
GA50 vs. DMSO10	−4.500	−21.76 to 12.76	No	ns	0.9533
GA50 vs. DMSO2	−6.000	−23.26 to 11.26	No	ns	0.8616
GA50 vs. DMEM	−10.75	−28.01 to 6.506	No	ns	0.3743
GA50 vs. Allantoin	−5.500	−22.76 to 11.76	No	ns	0.8985
GA400 vs. DMSO10	15.50	−1.756 to 32.76	No	ns	0.0910
GA400 vs. DMSO2	14.00	−3.256 to 31.26	No	ns	0.1481
GA400 vs. DMEM	9.250	−8.006 to 26.51	No	ns	0.5276
GA400 vs. Allantoin	14.50	−2.756 to 31.76	No	ns	0.1263
DMSO10 vs. DMSO2	−1.500	−18.76 to 15.76	No	ns	0.9997
DMSO10 vs. DMEM	−6.250	−23.51 to 11.01	No	ns	0.8409
DMSO10 vs. Allantoin	−1.000	−18.26 to 16.26	No	ns	> 0.9999
DMSO2 vs. DMEM	−4.750	−22.01 to 12.51	No	ns	0.9421
DMSO2 vs. Allantoin	0.5000	−16.76 to 17.76	No	ns	> 0.9999
DMEM vs. Allantoin	5.250	−12.01 to 22.51	No	ns	0.9147

The symbols * and **** are used to demonstrate the level of significance, indicating *P* values less than 0.05 and 0.0001, respectively

**Fig. 7:**
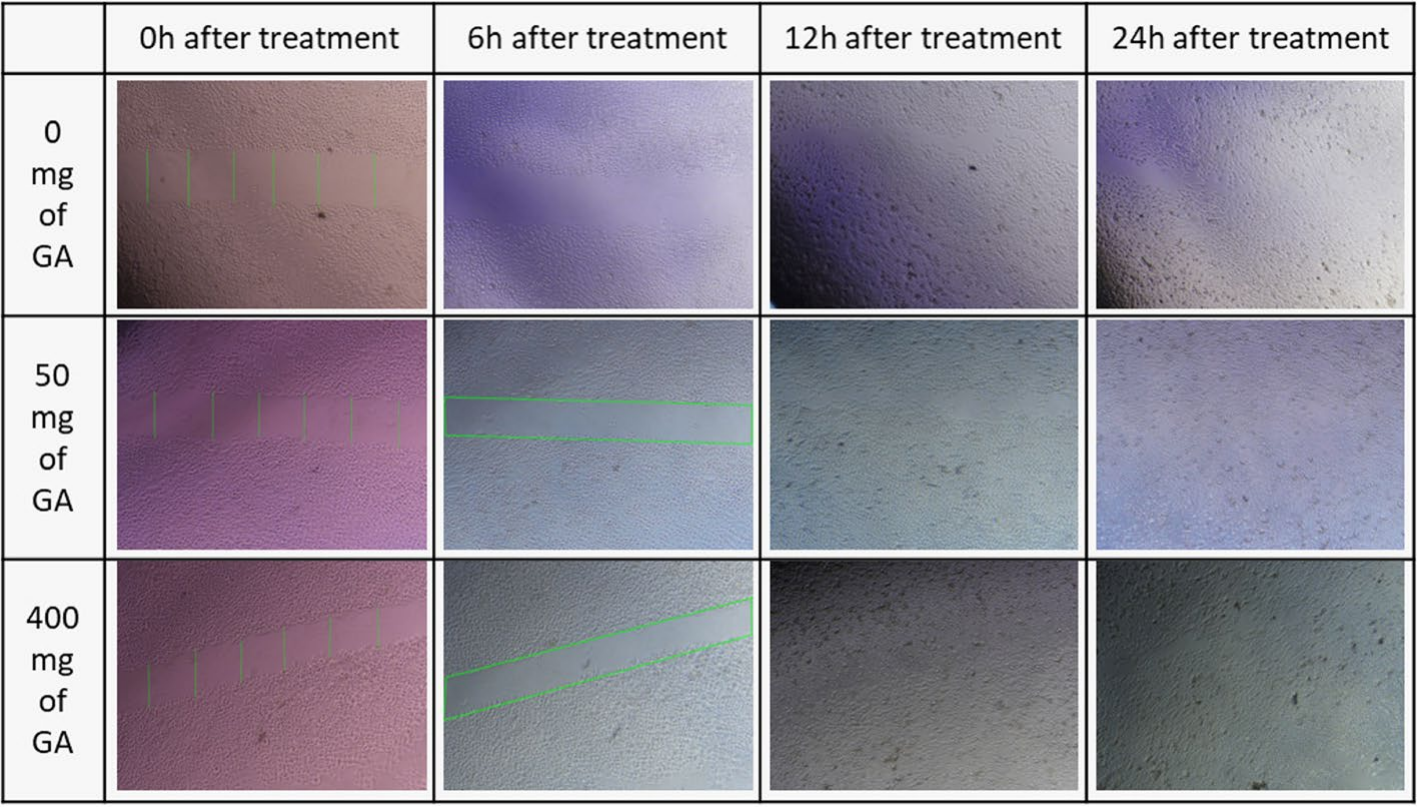
Microscopic Image of scratch and cell migration which is a main component of wound healing process. In this image, 0 mg/l (DMSO 2%), 50 (mg/ml), and 400 mg/ml of gallium aparine L. were used to treat the scratch on HUVEC cells

**Fig. 8:**
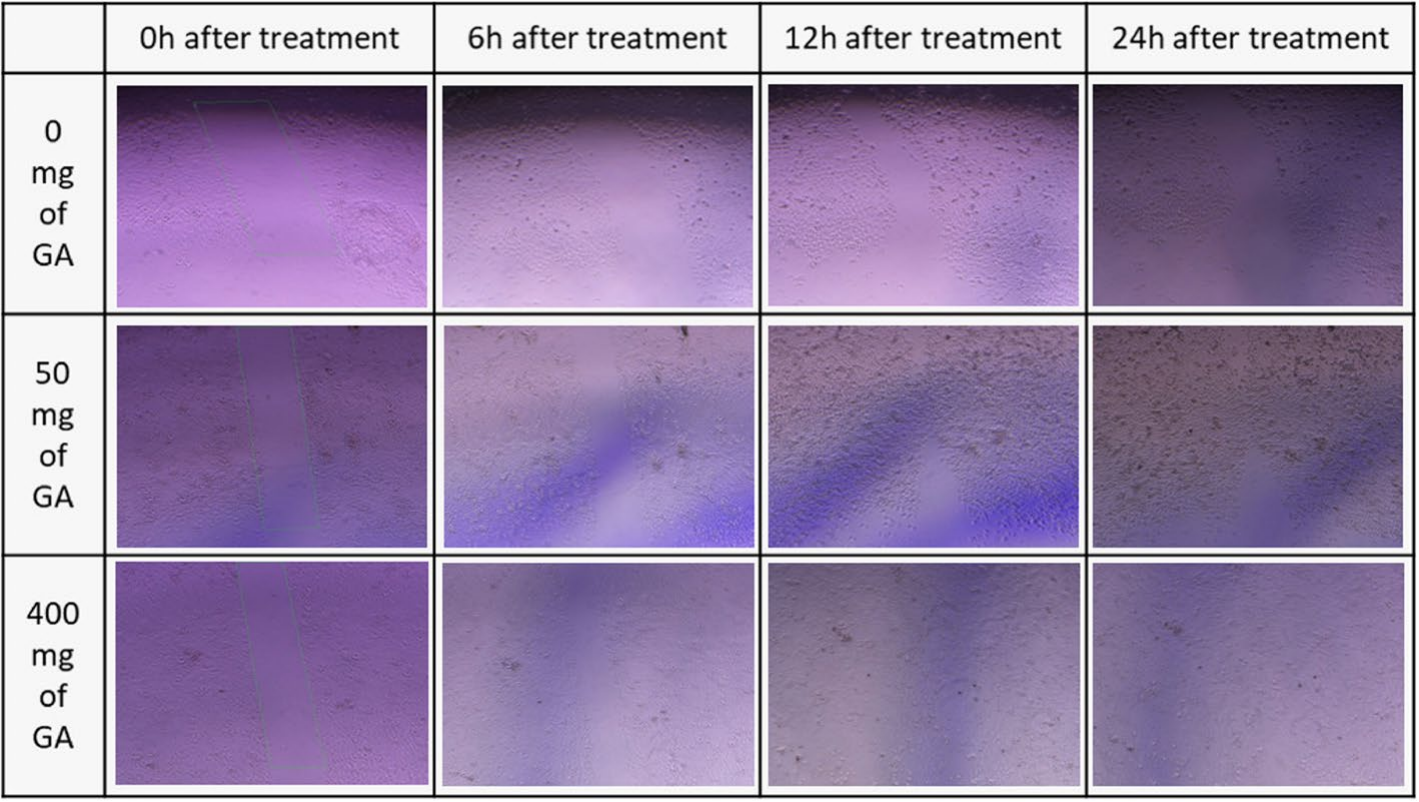
Microscopic Image of scratch and cell migration is the main component of the wound healing process. In this image, 0 mg/l (DMSO 2%), 50 (mg/ml), and 400 mg/ml of gallium aparine L. were used to treat the scratch on NHDF cells

Finally, the images of the scratch and the migration process are shown in Figs. 7 and 8.

## Discussion

**Fig. 9:**
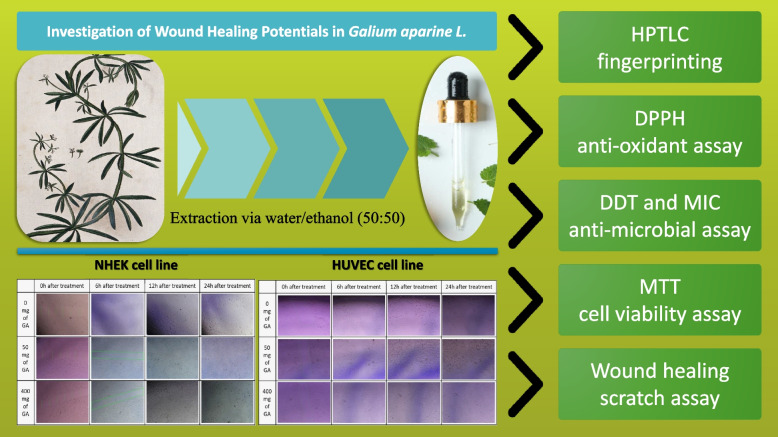
A graphical abstract of the experiment

Wound regeneration is directed by a chain of biological processes which are properly harmonized. Wound healing can generally occur by four coinciding but discrete steps, including hemostasis, inflammation, proliferation, and ultimately, wound remodeling, which may be affected by various endogenous and exogenous factors [38–40]. A large number of the population is inflicted by various types of acute and chronic wounds annually, and extensive costs are reported to be imposed on patients and healthcare systems, which indicates the importance of proper treatment’s role in the subtraction of patient complications and remedies charge [41]. Medicinal plants and natural products have long been famous and trusty remedies for different disorders; the application of herbal resources for wound treatment may be one count of these recommendations [42–44]. In the current experiment, we have evaluated the wound healing activity of *Galium aparine* extract by an in vitro analytical approach; and the antibacterial, antioxidant, and wound closure enhancing properties of the extract have been assessed through standard methods (see graphical abstract in Fig. 9). Previous phytochemical experiments on GA by various analytical methods revealed the presence of different compounds such as hydroxycinnamic acid derivatives, flavonoids, polyphenols carotenoids, iridoids, tannins, saponins, alkaloids, and some other components [45–49]. In this study, our HPTLC fingerprint results confirmed the presence of phenols, flavonoids, tannins, plant acids, ergot alkaloids, flavonoids, anthraquinones, terpenoids, sterols, salicin, lipophilic compounds, saponins, and iridoids which demonstrate that our result ties well with previous studies. Despite the regulatory effect of reactive oxygen species (ROS) in the wound healing process, excessive generation of ROS or impaired ROS elimination ends up in oxidative impairment such as lipid peroxidation, DNA breakage, and enzyme inactivation, which all lead to delayed or non-healing wounds [50–52]. Several investigations confirmed the efficacy of antioxidant therapy [53]. In previous experiments, GA was found as a scavenger of DPPH, ABTS, hydroxyl, hydrogen peroxide, NO species, and superoxide radicals [18, 54]. In the present study, we assessed the radical scavenging capability of different concentrations of GA extract by DPPH assay; the results indicated antioxidant activity and radical scavenging. Earlier investigations evaluated the cytotoxic effect of GA methanolic (MeOH) extract. Some concentrations have been reported to be cytotoxic for MCF-7 and MDA-MB-231 human breast cancer, but no cytotoxicity was observed for the MCF-10A cell line [55]. We examined HUVEC and NHDF cell lines viability treated with GA extract. Interestingly, the concentrations used did not exhibit a toxic effect in exposure to fibroblasts or endothelial cells but promoted cell viability. By comparing the results of former experiments, we hoped to determine the antibacterial activity of the extract. Therefore, we assessed the inhibitory effect of the prepared extract on *S. aureus*, and *E. coli*, which have been reported as two common species isolated from wounds, by detection rates around 37 and 6%, and *S. epidermidis* as normal resident microbiota of the human skin, which its biofilm formation is a significant complication [56, 57]. The results of our study showed significant antibacterial activity of GA extract against *S. aureus*, *E. coli,* and *S. epidermidis*. In 2016 Sharifi-Rad et al. found *S. aureus* and MRSA highly susceptible to GA extract [58]. In another study, the antibacterial activity of GA stem extract has been assessed. *S. aureus* was reported to be most sensitive, and then *Pseudomonas aeruginosa* exhibited lower susceptibility; *E. coli* was resistant though [59]. In another experiment, Galium L. species lipophilic complexes showed an inhibitory effect on *S. aureus, E. coli, Pseudomonas aeruginosa, Bacillus subtilis, and Proteus vulgaris* [60]. Regarding results obtained from experiments conducted before and present study results concerning the antibacterial effect of GA approximately were in line.

The application of GA for chronic ulcers has been investigated before [61]. Our scratch assay results proved the wound healing efficacy of GA extract, and notably, it was more efficient than allantoin as a well-known healing agent. Consequently, all this evidence may be a promising finding for GA application as a natural healing agent.

Considering the promising wound-healing effects of GA presented in this study, further research is necessary to prove our results and lead to the preparation of wound-healing products. In this case both conventional formulations such as wound healing ointments or herbal wound dressings [62, 63]. As well as novel formulations such as chitosan-based hydrogels and electrospun nanofibers [64–66] are important. Also, a comprehensive investigation into the phytochemical profile of GA extract is crucial. Unraveling the specific compounds responsible for wound healing properties of GA will not only deepen our understanding of its mechanism of action but also open doors for targeted isolation of these bioactive components and utilization of its wound healing activity. Additionally, functional investigation of the wound-healing properties of GA extract the in-vivo is necessary and animal models can be suggested to investigate the wound-healing effect of GA before designing any clinical trials. Such studies can be guided by our study and are essential to harness the full therapeutic potential of GA extract in the field of wound healing.

## Conclusion

Altogether, the present study results and other data available in the literature strongly suggest that GA extract may be beneficial to be applied as a wound-healing agent. Our data provide support for the antioxidant, antibacterial, and wound closure-enhancing properties of GA extract. In conclusion, GA can be used for subsequent additional in vitro, in vivo, and clinical studies to evaluate the wound healing potential of this herbal medicine from different aspects more practically.

## Data Availability

The datasets generated and analyzed during the current study are not publicly available due to institutional restrictions but are available from the corresponding author upon reasonable request.

## Abbreviations

GA: *Galium aparine L.*
HPTLC: High-performance thin-layer chromatography
TLC: Thin-layer chromatography
MIC: Minimum Inhibitory Concentration Test
DPPH: 2,2-Diphenyl-1-picrylhydrazyl
HUVEC: Human umbilical endothelial cell
NHDF: Normal human dermal fibroblasts
DMEM: Dulbecco’s Modified Eagle Medium
DMSO: Dimethyl sulfoxide
SD: Standard deviation
ROS: Reactive oxygen species
IZ: Inhibition zone
